# Regional Cardiac Pacing: Clarifying Learning‐Curve and Benchmarking Evidence

**DOI:** 10.1002/clc.70508

**Published:** 2026-11-09

**Authors:** Muhammad Safwan, Saman Naz, Abdihamid Hirsi Abdulle

**Affiliations:** ^1^ Saidu Medical College Swat Khyber Pakhtunkhwa (KPK) Pakistan; ^2^ Faculty of Medicine and Surgery Somali National University Mogadishu Banaadir Somalia

**Keywords:** cardiac pacing, cumulative sum analysis, healthcare decentralization, learning curve

To the editor,

1

We read with interest the study by Pikta et al. describing a mentored regional pacemaker program in Latgale [1]. Expanding access to pacing for geographically remote older adults is an important equity objective, and the authors' transparent account of adverse‐event‐driven procedural adaptations is commendable. However, several reporting and analytical issues require clarification before the learning‐curve and benchmarking conclusions can be reliably interpreted.

First, phase‐specific complication reporting is internally inconsistent. Figure 2 shows five, three, and two complications during the acquisition, stabilization, and expertise phases, respectively. Although the Results text attributes Phase 3 complications to complex anatomy, Table 2 assigns seven events to Phase 1, three to Phase 2, and none to Phase 3. Clarification is needed regarding the adjudicated event counts and whether phase‐specific rates were calculated using events or affected patients as numerators [1].

Second, the information provided does not permit evaluation of the reported *p* < 0.05 reduction between the acquisition and expertise phases. The statistical test, prespecified performance target, acceptable and unacceptable failure rates, and decision limits are not reported. Thus, Figure 2 demonstrates temporal variation in observed events but does not, as reported, substantiate a formal competency threshold or the designation of expertise after approximately 100 cases. Contemporary cumulative sum (CUSUM) learning‐curve studies prespecify procedural targets before identifying proficiency transitions [2].

Third, the reported historical benchmark comparison cannot substantiate assertions of superiority or non‐inferiority. Figure 3A labels the expertise cohort as “*n* = 116,” whereas the stated 3.4% expertise‐phase rate corresponds to two events among 58 procedures. Furthermore, neither a confidence interval nor a non‐inferiority margin is reported. These discrepancies should be reconciled, and the comparison should remain descriptive unless a prespecified non‐inferiority analysis is provided [1].

Finally, the reported procedural volume requires clarification. The cohort comprised 173 procedures over 2 years performed by two primary local operators, equivalent to approximately 43 procedures per operator‐year if caseload was shared equally. This does not readily reconcile with the reported annual volume exceeding 75–80 cases for each operator. Operator‐specific case counts and complication rates would allow verification of the volume claim and strengthen interpretation of the learning curve [1].

We respectfully raise these points to strengthen interpretation of the findings. Reconciling the reported data and clearly specifying the analytical framework would enhance the study's reproducibility and its value for centers planning the safe decentralization of cardiac pacing services.

## Author Contributions

M.S. and S.N. contributed to the conception of the letter, literature review, drafting, and critical revision of the manuscript. A.H.A. assisted in reviewing and editing the final version. All the authors meet the ICMJE authorship criteria, approved the final version, and agreed to be accountable for all aspects of the work, ensuring the accuracy and integrity of the data and interpretation.

## Funding

The authors have nothing to report.

## Ethics Statement

This Letter to the Editor does not report research involving human participants, animals, or identifiable human data and therefore did not require approval from a Research Ethics Committee/Institutional Review Board.

## Conflicts of Interest

The authors declare no conflicts of interest.

## Data Availability

No datasets were generated or analyzed during the current study.
